# Insights into the impact of *flhF* inactivation on *Campylobacter jejuni* colonization of chick and mice gut

**DOI:** 10.1186/s12866-018-1318-1

**Published:** 2018-10-22

**Authors:** Fangzhe Ren, Xiaofei Li, Haiyan Tang, Qidong Jiang, Xi Yun, Lin Fang, Pingyu Huang, Yuanyue Tang, Qiuchun Li, Jinlin Huang, Xin-an Jiao

**Affiliations:** 1 Jiangsu Key Lab of Zoonosis/Jiangsu Co-Innovation Center for Prevention and Control of Important Animal Infectious Diseases and Zoonoses, Yangzhou University, 48 East Wenhui Road, Yangzhou, 225009 Jiangsu China; 2 Key Laboratory of Prevention and Control of Biological Hazard Factors (Animal Origin) for Agrifood Safety and Quality, Ministry of Agriculture of China, Yangzhou, 225009 Jiangsu China; 3 Joint International Research Laboratory of Agriculture and Agri-product Safety, Ministry of Education of China, Yangzhou, 225009 Jiangsu China

**Keywords:** *flhF*, Colonization, *Campylobacter jejuni*

## Abstract

**Background:**

*Campylobacter jejuni* (*C. jejuni*) is a leading cause of foodborne gastroenteritis worldwide. This bacterium lacks many of the classical virulence factors, and flagellum-associated persistent colonization has been shown to be crucial for its pathogenesis. The flagellum plays a multifunctional role in *C. jejuni* pathogenesis, and different flagellar elements make diverse contributions. The *flhF* gene encodes the flagellar biosynthesis regulator, which is important for flagellar biosynthesis. In this study, the influence of *flhF* on *C. jejuni* colonization was systematically studied, and the possible mechanisms were also analyzed.

**Results:**

The *flhF* gene has a significant influence on *C. jejuni* colonization, and its inactivation resulted in severe defects in the commensal colonization of chicks, with approximately 10^4^- to 10^7^-fold reductions (for NCTC 11168 and a *C. jejuni* isolate respectively) observed in the bacterial caecal loads. Similar effects were observed in mice where the *flhF* mutant strain completely lost the ability to continuously colonize mice, which cleared the isolate at 7 days post inoculation. Characterization of the phenotypic properties of *C. jejuni* that influence colonization showed that the adhesion and invasion abilities of the *C. jejuni flhF* mutant were reduced to approximately 52 and 27% of that of the wild-type strain, respectively. The autoagglutination and biofilm-formation abilities of the *flhF* mutant strain were also significantly decreased. Further genetic investigation revealed that *flhF* is continuously upregulated during the infection process, which indicates a close association of this gene with *C. jejuni* pathogenesis. The transcription of some other infection-related genes that are not directly involved in flagellar assembly were also influenced by its inactivation, with the flagellar coexpressed determinants (Feds) being apparently affected.

**Conclusions:**

Inactivation of *flhF* has a significant influence on *C. jejuni* colonization in both birds and mammals. This defect may be caused by the decreased adhesion, invasion, autoagglutination and biofilm-formation abilities of the *flhF* mutant strain, as well as the influence on the transcription of other infection related genes, which provides insights into this virulence factor and the flagellum mediated co-regulation of *C. jejuni* pathogenesis.

**Electronic supplementary material:**

The online version of this article (10.1186/s12866-018-1318-1) contains supplementary material, which is available to authorized users.

## Background

*Campylobacter jejuni* is the most common cause of foodborne gastroenteritis in humans worldwide, and infections caused by this microbe often leads to diarrhea and sometimes to a severe inflammatory response with clinical symptoms of fever, abdominal cramping, bloody stools and other symptoms [1, 2]. *C. jejuni* is prevalent in the environment and can infect humans in a variety of ways [3, 4]. Although the incidence of *C. jejuni* infection is very high at present, causing it to receive increased attention [5], a complete understanding of the mechanisms of *C. jejuni*-associated disease is still an ongoing effort [3]. An analysis of the genome sequence of *C. jejuni* revealed that the cytolethal distending toxin (CDT) is the only toxin produced by this bacterium. Many other classical virulence factors that are common to other pathogens are missing in the *C. jejuni* genome [6]. It has now been shown that colonization in the host intestinal tract and persistence to a sufficient load is crucial for *C. jejuni* pathogenesis [3, 7, 8].

Intestinal colonization by *C. jejuni* is influenced by multiple factors, including the capsule, glycosylation system, catabolism of L-serine, cytochrome c peroxidase, transport systems, putative adhesins, chemotaxis and flagellar motility [9–16]. The flagellum is particularly important among these factors [17]. *C. jejuni* produces a single flagellum at one or both poles, which not only provides chemotactic motility for migration to replicative niches but also helps the bacteria penetrate the mucus that covers the epithelial cells. In addition, the flagellum is involved in interactions with and invasion of the host epithelium, the secretion of invasion antigens, and the evasion of the innate immune system, is the essential virulence and colonization factor of *C. jejuni* [6, 15, 18–20]. The flagellum is a multicomponent organelle composed of a basal body, motor, rod, hook and flagellin filament. It appears that the *C. jejuni* flagellar substructures, flagellin glycosylation, motility and secreted invasion antigens are all required for host colonization but are not the only determinants [9, 16, 19, 21–23]. There are over 50 flagellum-related genes in the *C. jejuni* genome, and their corresponding contributions to colonization are also diverse [15, 16, 18, 22–25]. Considering the multifunctional role played by flagella during *C. jejuni* pathogenesis [26], a comprehensive understanding of their virulence potential is urgently needed.

The importance of FlhF for flagellar biosynthesis has been observed for several pathogens (primarily for the polarly flagellated bacteria) in recent years [27–33]. In *C. jejuni*, the inactivation of *flhF* has been observed to inhibit the synthesis of flagella and motility in previous studies [34, 35], but its contribution to *C. jejuni* colonization has not yet been characterized. In this study, the influence of *flhF* on *C. jejuni* colonization was systematically evaluated both in the reference strain and in an isolate. The results showed the significant effect this gene has in the host colonization process. The possible factors that may contribute to the colonization defect were assayed, including the flagella-associated abilities such as adherence and invasion to the host cells, autoagglutination (AAG) and biofilm formation. The mechanism of by which FlhF influences host colonization was also explored at the gene expression level. We observed that *flhF* is continuously upregulated during the cell infection process, and in addition to its major role in the flagellar system, some other infection-related genes were observed to be downregulated in the *flhF* mutant strain. The results of this study provide a reference for the further analysis of this virulence factor and investigations of the mechanism of flagella mediated co-regulation of pathogenesis in this bacterium.

## Methods

### Bacteria and cell culture

*C. jejuni* strains were grown on *Campylobacter* blood-free selective agar containing charcoal cefoperazone deoxycholate (CCDA) (Oxoid, Basingstoke, UK) or in Mueller-Hinton (MH) broth (BD Biosciences, Sparks, MD, USA) in a microaerophilic environment (5% O2, 10% CO2, and 85% N2) and incubated at 42 °C. The *C. jejuni* isolate used in this study was isolated from the anal swabs of a monkey with diarrhea and was further identified using multiplex PCR (polymerase chain reaction) (Additional file 1: Figure S1) [36]. Appropriate antibiotics (50 μg/ml kanamycin, 20 μg/ml chloramphenicol, 10 μg/ml rifampicin, 6 μg/ml polymyxin B, 10 μg/ml trimethoprim, 100 μg/ml cycloheximide, 40 μg/ml vancomycin, 20 μg/ml amphotericin B or 60 μg/ml cefoperazone) were added to the medium as required. Inactivation of *flhF* and complementation of the mutant in *C. jejuni* NCTC 11168 and in the isolate were performed as previously described [34]. Human colon cancer cell line (Caco-2) was acquired from American Type Cell Culture (ATCC) and routinely cultured in Dulbecco’s minimal essential medium (DMEM) supplemented with 10% fetal bovine serum (FBS) and maintained in a humidified 37 °C atmosphere containing 5% CO2.

### Colonization assay

Chick and mice colonization assays were largely performed as described in previous studies [16, 24, 37]. Briefly, *C. jejuni* strains were harvested in phosphate buffered saline (PBS), and 1 × 10^8^ colony-forming units (cfu) of bacteria were subsequently orally inoculated into 1-day-old *Campylobacter*-free Leghorn chicks obtained from the Jiangsu Institute of Poultry Science. Chicks were randomly allocated into three groups and sacrificed by CO2 asphyxiation at 1 and 7 days post inoculation. For each chick, the intestinal tract was collected, separated following gentle extrusion of the lumenal contents, weighed, homogenized, serially diluted and plated on selective CCDA agar (containing rifampicin, polymyxin B, trimethoprim, cycloheximide, vancomycin, amphotericin B and cefoperazone) to count the bacteria load. Six-week-old C57/BL6 mice were acquired from the Comparative Medicine Center of Yangzhou University. The mice initially received 200 μl of a sodium bicarbonate solution (50 mg/ml), after which 1 × 10^8^ cfu of bacteria were used to infect mice by orogastric inoculation 15–30 min later. The mice were sacrificed by cervical dislocation at the designated time point, and the colonization levels were evaluated using selective media containing amphotericin B and cycloheximide as described above.

### Growth and biochemical tests

Overnight cultures of *C. jejuni* were harvested and diluted in MH broth to a starting optical density at 600 nm (OD600) of 0.08. Two-milliliter aliquots of the suspension were transferred into the wells of a 12-well plate and incubated at 42 °C and 120 rpm under a microaerophilic atmosphere. Samples were collected at indicated intervals and the OD600 values were determined to compare the growth of the different strains. Biochemical testing was performed as described by Valenza et al. [38]. Bacteria were harvested in a 0.45% NaCl solution and the turbidities were adjusted to an equivalent of a McFarland standard of 3. The suspensions were inoculated into a Neisseria-Haemophilus (NH) identification card, which was automatically analyzed by a VITEK 2 microbiology system (bioMérieux, France).

### Cell adhesion and invasion assay

Adhesion and invasion assays were performed as described previously [39]. Caco-2 cells were first seeded into a 24-well plate at a density of 1 × 10^5^. After the cells incubated for 18 h, *C. jejuni* that had grown for 20 h were harvested and resuspended in DMEM, and 1 ml suspensions containing 1 × 10^7^ cfu were then inoculated into each well. The plates were centrifuged at 600×*g* for 5 min to enhance the contact between bacteria and eukaryotic cells. For bacterial adhesion assays, cells were incubated for 2 h, washed three times with PBS to remove the unattached bacteria, and then were lysed in 1 ml of PBS containing 0.1% TritonX-100, with serial dilutions of the suspensions subsequently spread onto CCDA plates to quantify the number of bacteria. To assess the number of invaded bacteria, the inoculated cells were first incubated for 2 h, washed once with DMEM and then were incubated in DMEM containing gentamicin (100 μg/ml) for another 1 h. The extracellular bacteria were killed during this period, and the number of invaded bacteria was determined as described above.

### Autoagglutination assay

The autoagglutination assay was performed as previously described [23]. *C. jejuni* strains were grown for 20 h prior to use, after which each strain was suspended in PBS and adjusted to an OD600 of 1.0. Five-milliliter aliquots of the suspension were transferred into a series of sterile glass tubes (13 × 100 mm), which were incubated under microaerophilic conditions for a predetermined time. After incubation, 200 μl of the bacterial suspension was carefully removed from the top of each tube, and the OD600 was measured to evaluate the degree of AAG. Bacterial cells that strongly agglutinate will fall to the bottom of the tube, leading to a decreased OD600 value.

### Biofilm-formation assay

The ability of *C. jejuni* strains to form biofilms was measured by crystal violet staining, using a method described by Naito et al. with some modifications [40]. Briefly, bacteria cultured for 20 h were harvested and diluted in MH broth to an OD600 of 0.1, after which 200 μl of each suspension was inoculated into 96-well polypropylene plates and incubated at 42 °C under microaerobic conditions. After incubating for 48, 60, 72, 84 and 90 h, the plates were washed with PBS and dried at 60 °C for 30 min. Next, 200 μl of a 1% crystal violet solution was added to each well to stain the biofilms that had formed. After staining at room temperature for 30 min, the plates were rinsed thoroughly with PBS three times to remove the unbound crystal violet. The plates were subsequently dried at 60 °C for 15 min, and the biofilms were quantified by determining the OD570 values after the remaining crystal violet was solubilized in a solution composed of 30% methanol and 10% acetic acid.

### RNA isolation and quantitative real-time PCR

Quantitative real-time PCR (qRT-PCR) was performed as described previously [34]. *C. jejuni* was cultured for designated time, after which the RNA was extracted using an RNeasy plus mini kit (Qiagen, Hilden, Germany) according to the manufacturer’s instructions. The isolated RNA was then used for cDNA synthesis using an RT reagent Kit (TaKaRa, Dalian, China). qRT-PCR was performed with a ABI PRISM 7500 Real-Time PCR System (Applied Biosystems, Foster City, CA, USA) using the FastStart Universal SYBR Green Master (ROX) (Roche Diagnostics, GmbH, Germany). The primers used for qRT-PCR are listed in the Additional file 1: Table S1. The *cj0402* gene (*glyA*) was used as an endogenous control to evaluate the relative expression of the target genes.

### Gene collection and microarray analysis

The genes required for *C. jejuni* adhesion/invasion and colonization that were not directly involved in flagellar assembly were identified and are listed in Additional file 1: Table S2, and this information was primarily obtained from previously published reports. The relative mRNA expression of the selected genes in the *C. jejuni flhF* mutant strain versus the wild-type strain were acquired and analyzed using our previous whole transcriptome microarray data [34], which has been submitted to the NCBI Gene Expression Omnibus (GEO) database with the accession number GSE 87852. The fold change in gene expression was graphically presented in a heatmap that was generated using the Hemi software, with different colors representing the degree of variation in expression [41].

### Statistical analysis

The data are presented as the means ± standard deviation (SD) from three independent experiments. Student’s t-test was used to assess the significance of the observed differences (*P* < 0.05 *, and *P* < 0.01 **).

## Results

### Inactivation of *flhF* in *C. jejuni* dramatically influences its colonization in both chicks and mice

**Fig. 1:**
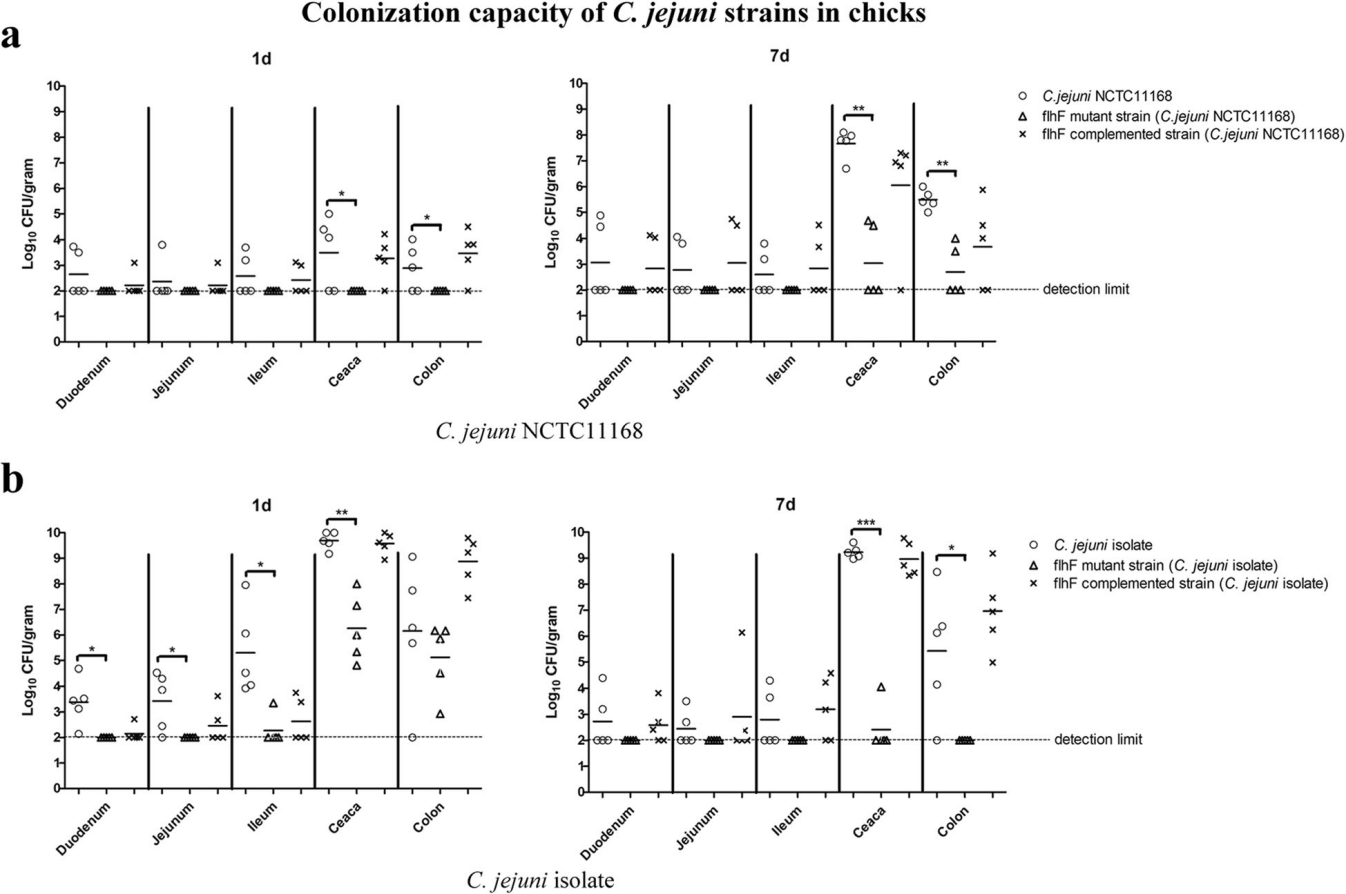
Colonization capacity of *C. jejuni* strains in chicks. The influence of *flhF* on *C. jejuni* colonization in chicks was evaluated for both the reference strain NCTC 11168 (**a**) and a *C. jejuni* isolate (**b**). The wild-type strain (hollow circle), the *flhF* mutant strain (hollow triangle) and the *flhF* complemented strain (cross) are represented by each sample. Five chicks were used in each group, the mean values for each group are depicted by the horizontal bar and asterisks indicate significant differences (*P* < 0.05 *, and *P* < 0.01 **)

The importance of *flhF* on *C. jejuni* commensal colonization in chicks was systematically analyzed for both the reference strain NCTC 11168 and for a *C. jejuni* isolate. For NCTC 11168 (Fig. 1a), the *C. jejuni* bacterial load in the intestinal tract was relatively low 1 day after inoculation. However, we could not isolate the *flhF* mutant strain at this time point and a significant difference in colonization was observed compared to the wild-type strain in the large intestine (caeca and colon) (*P* < 0.05). On day 7, the *flhF* mutant strain still could not colonize the small intestines of chicks (duodenum, jejunum and ileum), and although it was present in the large intestines of some chicks, the bacterial load was significantly lower than that observed for NCTC 11168-infected group (*P* < 0.01).

A hyper-colonization phenotype was observed for the *C. jejuni* isolate, as the bacterial loads in the intestines of chicks infected with the same dose were notably higher than in those of NCTC 11168-infected chicks, thus the effect of *flhF* was further determined in this *C. jejuni* isolate. As shown in Fig. 1b, the *flhF* gene was observed to be important in the initial establishment of colonization, since at 1 day post infection, the number of recovered isogenetic *flhF* mutant was significantly lower than that observed for chicks infected with the wild-type strain for both the small intestine (*P* < 0.01) and the caeca (*P* < 0.05). On day 7, the *flhF* mutant strain was almost completely cleared from the chicks and could be found only in the caeca of one chick, while the wild-type *C. jejuni* isolate consistently colonized the large intestines of chicks with a high load (approximately 10^9^ cfu/g in the caeca). Complementation of *flhF* recovered this deficiency somewhat, both in *C. jejuni* NCTC 11168 and in the isolate, indicating that *flhF* significantly influences the commensal colonization of chicks.

**Fig. 2:**
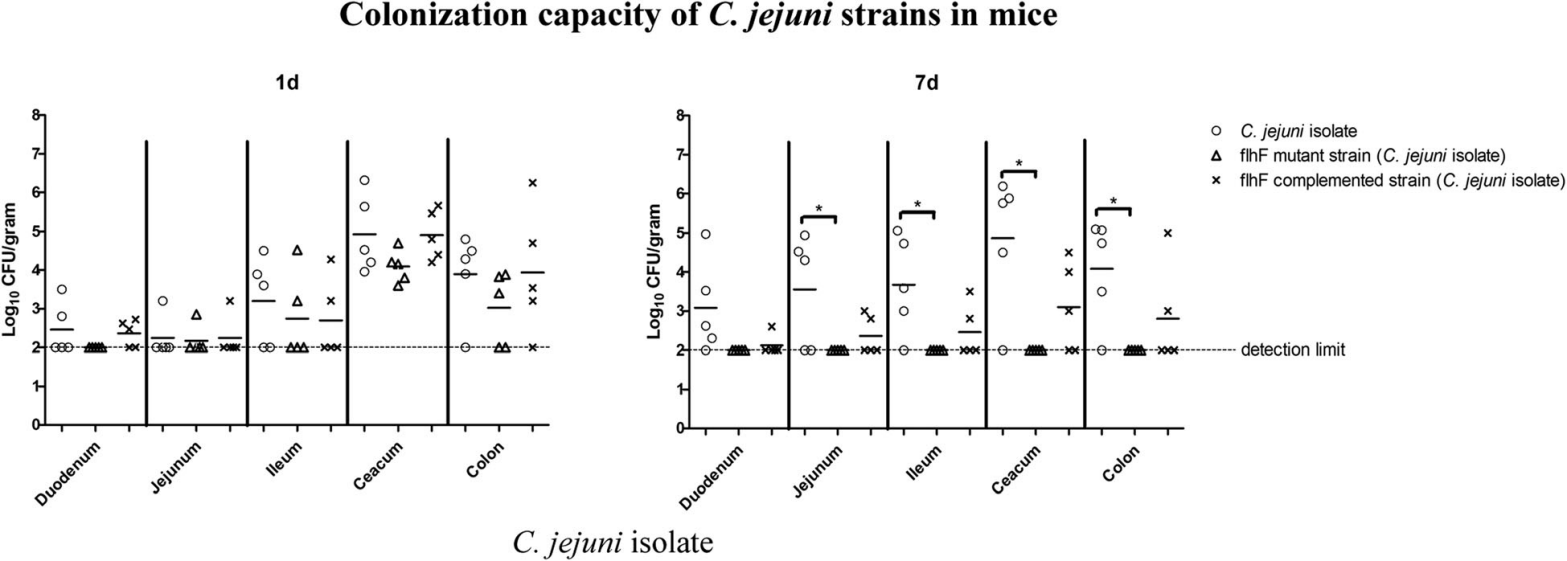
Colonization capacity of *C. jejuni* strains in mice. The influence of *flhF* on *C. jejuni* colonization in mice was evaluated in the *C. jejuni* isolate. The wild-type strain (hollow circle), the *flhF* mutant strain (hollow triangle) and the *flhF* complemented strain (cross) are represented by each sample. Five mice were used in each group, the mean values for each group are depicted by the horizontal bar and asterisks indicate significant differences (*P* < 0.05 *)

The mice were less susceptible to *C. jejuni* colonization than the chicks. Although the *C. jejuni* isolate was observed to stably colonize both animals, the reference strain NCTC 11168 failed to colonize the mice in our study. For the *C. jejuni* isolate (Fig. 2), the bacteria recovered from the intestinal tract of mice were notably less abundant than those recovered from the chicks. However, the results showed that *flhF* does not greatly influence the initial colonization of mice. Although the average amount of the isolated *flhF* mutant was less than that of the wild-type strain, no significant differences were observed after 1 day post inoculation. On day 7, the *flhF* mutant strain could not be recovered from any regions of the intestines, while the wild-type strain continuously colonized the mice, with significant differences observed in the jejunum, ileum, caecum and colon (*P* < 0.01). These results suggested that inactivation of *flhF* resulted in the eventual elimination of the bacteria in mice.

### *flhF* does not influence the growth or primary biochemical characteristics of *C. jejuni*

**Fig. 3:**
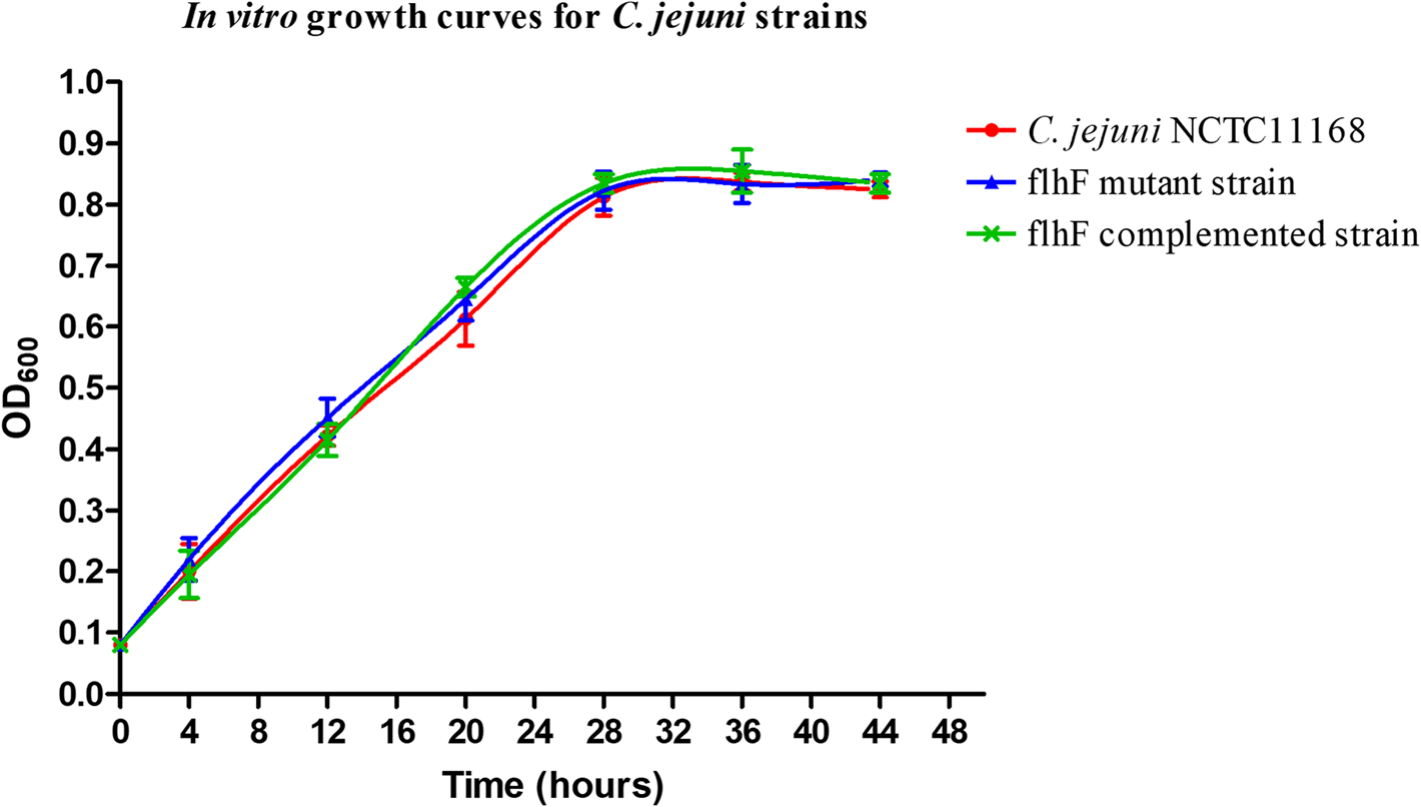
Growth curves of *C. jejuni* strains. *C. jejuni* NCTC 11168 and its derivatives were initially adjusted to an optical density at 600 nm (OD600) of 0.08, after which they were grown in MH broth under microaerophilic conditions with shaking at 42 °C, with 200 μl of samples were withdrawn at designated time point to examine growth. The wild-type strain (red circle), the *flhF* mutant strain (blue triangle) and the *flhF* complemented strain (green cross) are represented by each sample. The data are presented as the means ± standard deviation (SD) from three independent experiments

The observed growth curves indicated that the inactivation of *flhF* does not influence the normal growth of *C. jejuni*. When cultured in MH broth, the OD600 values for *C. jejuni* NCTC 11168, the *flhF* mutant and the complemented strains were nearly same at different time points and no significant differences were observed, suggesting that these strains have a similar growth rate (Fig. 3). The biochemical characteristics of the *C. jejuni* strains were analyzed using an NH card with a VITEK 2 compact instrument. The NH card contains 30 biochemical tests that includes five alkalization tests, 10 acidification tests, 11 glycosidase and peptidase tests and four miscellaneous tests. For the *C. jejuni flhF* mutant and the complemented strain, all the indexes tested were the same as the NCTC 11168 strain except for OPS (phenylphosphonate) (Additional file 1: Table S3), indicating that the primary biochemical characteristics of *C. jejuni* were unaffected in the *flhF* mutant.

### *C. jejuni flhF* mutant shows a decreased ability to infect cultured epithelial cells

**Fig. 4:**
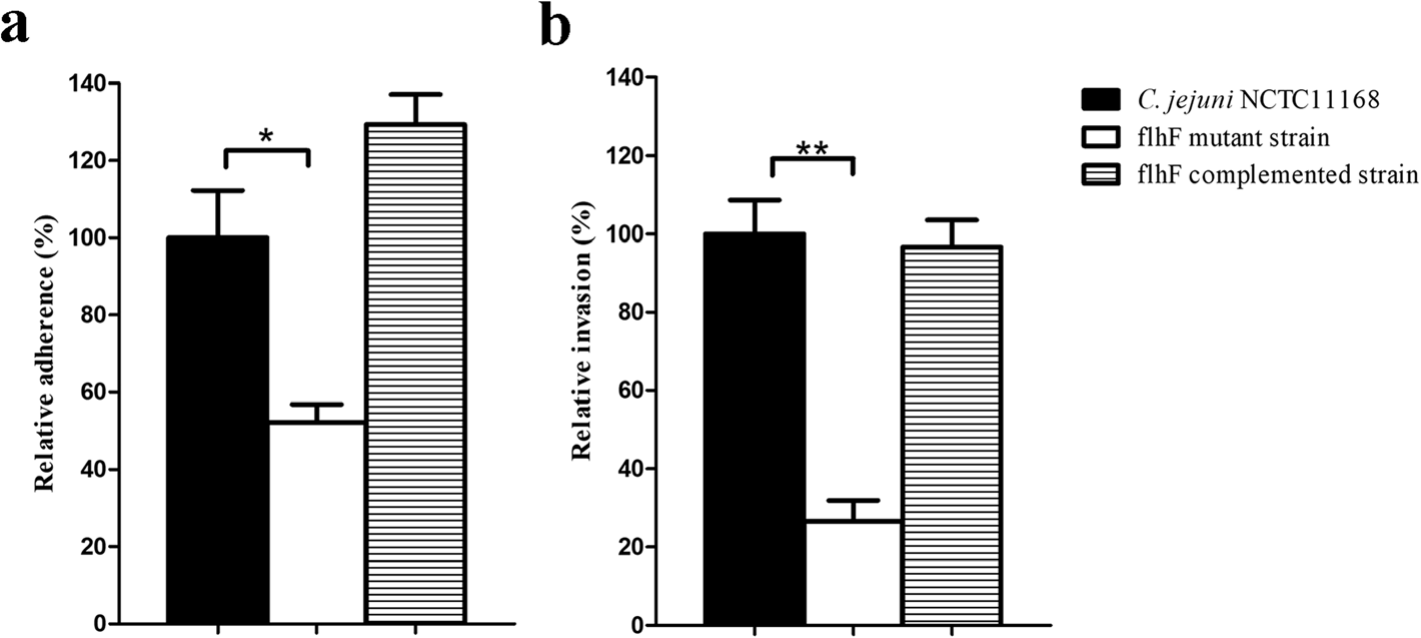
The ability of *C. jejuni* strains to adhere to and invade Caco-2 cells. Cells were infected with *C. jejuni* NCTC 11168 and its derivatives for 2 h. A comparison of the levels of adherence is presented as the values of *C. jejuni* mutant and complemented strains relative to that of the wild-type strain, which was set at 100% (**a**). Gentamicin was added to another plate of cells and incubated for another 1 h to enumerate the invaded bacteria (**b**). The data are presented as the means ± standard deviation (SD) from three independent experiments. Significant differences (*P* < 0.05 *, and *P* < 0.01 **) are indicated by asterisks

To characterize the infectivity of *C. jejuni* after inactivation of *flhF*, the ability of the strains to invade cultured epithelial cells was assessed. As shown in Fig. 4, inactivation of *flhF* significantly influenced the ability of *C. jejuni* to infect intestinal epithelial cells. Despite the effort made to minimize motility-dependent effects via centrifuging cells, the relative percentage of the *flhF* mutant strain to adhere and invade Caco-2 cells was 52 and 27%, respectively, compared to the NCTC 11168 strain. Complementation with *flhF* restored this defect in the mutant, and the infection ability of the complemented strain was nearly same as that observed for the wild-type strain (129 and 97% for adherence and invasion, respectively). These results suggest that the adherence to the epithelium and the subsequent invasion during *C. jejuni* infection is hampered by the inactivation of *flhF*.

### The autoagglutination and biofilm-formation abilities of *C. jejuni* are decreased by the inactivation of *flhF*

**Fig. 5:**
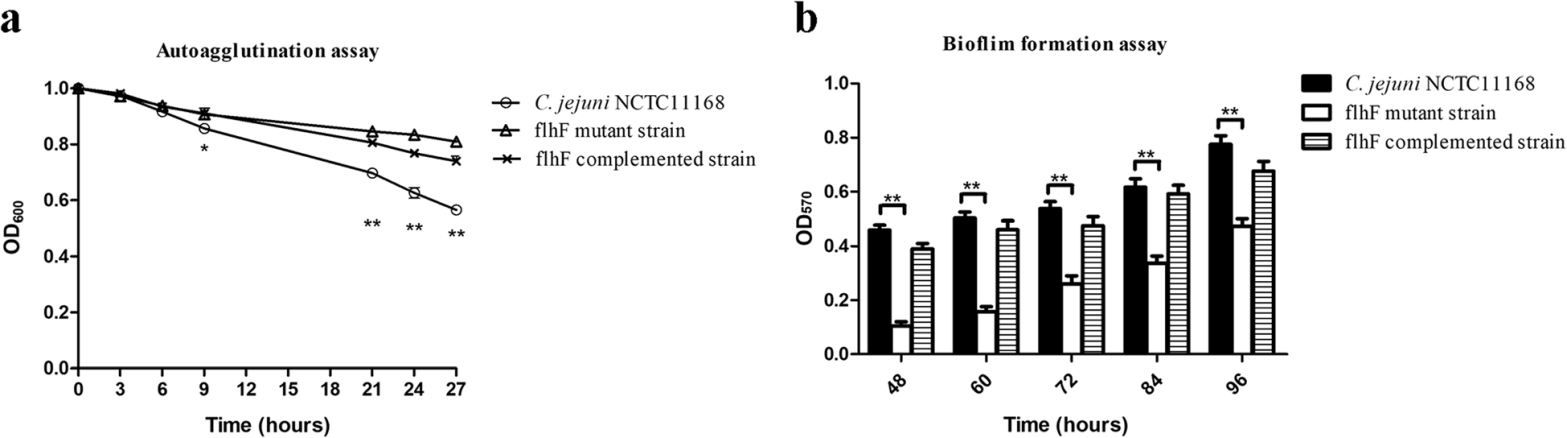
Autoagglutination and biofilm formation of *C. jejuni* strains. *C. jejuni* NCTC 11168 and its derivatives were cultured for the designated times. Strains that strongly agglutinate will fall to the bottom of the tube, and a 200 μl samples from the top of the suspensions were taken to determine the OD600 values. The autoagglutination abilities of the *C. jejuni* wild-type strain (hollow circle), the *flhF* mutant strain (hollow triangle) and the *flhF* complemented strain (cross) were compared (**a**). The formed biofilms were stained with crystal violet, and biofilm formation was quantitated by determining the OD570 value (**b**). The data are presented as the means ± standard deviation (SD) from three independent experiments. Significant differences (*P* < 0.05 *, and *P* < 0.01 **) are indicated by asterisks

The AAG ability of *C. jejuni* strains was compared after they were statically incubated for a short (3, 6, and 9 h) and long (21, 24, and 27 h) time spans (Fig. 5a). The AAG phenotype of each tested strain became more prominent over time. During the first 6 h, no significant difference in the AAG ability was observed between the *C. jejuni* NCTC 11168 and *flhF* mutant strains. The influence of *flhF* on *C. jejuni* AAG became noticeable after the strains were incubated for 9 h. The measured OD600 of *C. jejuni* NCTC 11168 was significantly lower than that of the *flhF* mutant strain (*P* < 0.05), and this effect increased over time, as the observed difference was more significant at 21–27 h (*P* < 0.01).

The biofilms formed by the strains were quantified after being statically cultured for 48–96 h. As shown in Fig. 5b, the biofilms formed for each strain continuously increased over time, but the biofilm-forming potential was different between the strains. The *flhF* mutant strain showed a defect in biofilm formation, as the OD570 values of the tested strains across all assayed time points were significantly decreased compared to those observed for the NCTC 11168 strain (*P* < 0.01).

The biofilm-formation defect was partially restored after complementation of *flhF*, the AAG ability was also increased somewhat in the complemented strain. The detected values for the biofilms formed and AAG ability of the complemented strain were all higher than the mutant at all the assayed time points, although these values did not reach the level of the wild-type strain.

### *flhF* is continuously upregulated during *C. jejuni* infection and influences the transcription of other infection-related genes

**Fig. 6:**
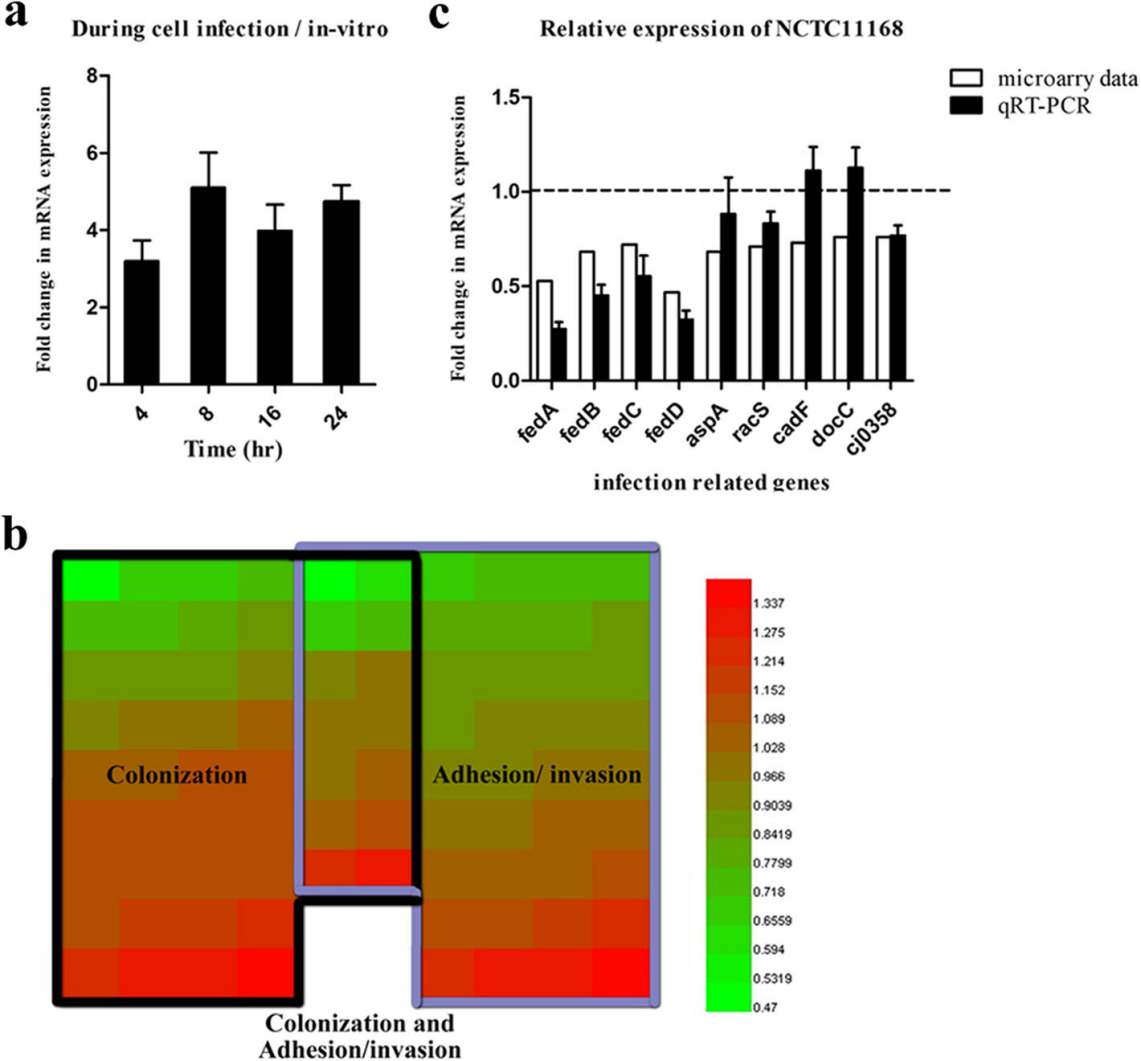
Analysis of relative mRNA expression. *C. jejuni* NCTC 11168 was grown in vitro and in Caco-2 cells. The *flhF* gene was observed to be continuously upregulated during the Caco-2 cell infection process. Each column represents the relative mRNA expression of *flhF* during the cell infection process to the in vitro group. The data are presented as the means ± standard deviation (SD) from three independent experiments (**a**). Heatmap presentation of relative mRNA expression of other infection-related genes not directly involved in flagellar assembly. Red and green in the heatmap represent gene upregulation and downregulation, respectively, in the *flhF* mutant relative to the wild-type strain, with more saturated colors representing a greater difference as indicated in the bar (**b**). Some representatives were selected for further qRT-PCR verification. Each column represents the relative mRNA expression of the *flhF* mutant strain relative to the *C. jejuni* NCTC 11168 strain. The data are presented as the means ± standard deviation (SD) from three independent experiments (**c**)

To analyze whether *flhF* is associated with *C. jejuni* infection in vivo, the NCTC 11168 strain was cultured in vitro and used to infect Caco-2 cell for 4, 8, 16 or 24 h, respectively. The RNA was extracted from samples at each time point, and a qRT-PCR assay was performed to compare the relative expression of *flhF* at the different time points. Compared to the in vitro groups, *flhF* was observed to be upregulated during the infection process at all the assayed time points. The observed fold-changes in expression were all over 3, and specifically for the 4, 8, 16 and 24 h time points were increased by 3.19-, 5.10-, 3.98- and 4.74-fold, respectively (Fig. 6a). This result suggests that *flhF* is a virulence-associated gene that may have a continuous role during *C. jejuni* infection.

The genes not directly involved in flagellar assembly but that were reported to influence *C. jejuni* adhesion, invasion and colonization were summarized (Additional file 1: Table S2) and their relative expression after inactivation of *flhF* was analyzed using our previous microarray data. As shown in Fig. 6b, both the colonization- (40%, 20/50) and adhesion/invasion- (50%, 25/50) related genes were downregulated after the inactivation of *flhF*. The average influence on adhesion/invasion was slightly higher than that observed for colonization. Some genes that could influence both the adhesion/invasion and colonization ability of *C. jejuni* were identified (the overlapping genes are presented in the middle of the heatmap), and their average transcription was observed to be decreased more than that of the other two groups. Some genes were selected for further qRT-PCR verification. In addition to *cadF* and *docC*, which were slightly upregulated in the qRT-PCR assay (Fig. 6c), all the other assayed genes showed the same trends as the microarray, where the transcription of *fedA*, *fedB*, *fedC* and *fedD* (Feds) were apparently affected. Thus, besides the major role *flhF* plays in the flagellar system, it may also affect *C. jejuni* infection in other ways, but the underlying mechanism still requires further investigation.

## Discussion

The efficient establishment of colonization is a prerequisite for *C. jejuni* pathogenesis. It was originally hypothesized that motility plays an essential role in *C. jejuni* colonization, as only motile strains could be recovered from human volunteers [42]. Subsequent studies revealed that flagella are an important colonization factor, as intact and motile flagella are required for efficient colonization [43]. The flagellar filament accounts for the largest proportion of the synthesized flagella and was the earliest studied flagellar substructure. The *C. jejuni* flagellar filament is composed of two almost identical flagellin proteins. Flagellin A is predominantly responsible for the intact and fully motile flagellation phenotype and has a specific contribution to *C. jejuni* colonization regardless of the effects observed by a defect in motility or inactivation of *flaB* [44]. Although Wassenaar et al. suggested that flagellin A, rather than motility, is essential for optimal colonization [44]. However, the role of motility cannot be ignored, as the mutation of *pflA*, which results in nonmotile bacteria with paralyzed flagella, also influences *C. jejuni* colonization [16].

Subsequent studies focused on the role of other flagellar elements. For the flagellar biosynthesis regulators RpoN (σ54) and FliA (σ28), mutation of either of these regulators resulted in an apparent attenuation for caecal colonization in chicks, but the loss of *fliA* had a more obvious effect than that of *rpoN*, despite its lesser influence on *C. jejuni* motility [18]. FlgR is a σ54-dependent response regulator that was observed to decrease the *C. jejuni* colonization ability by 10- to 500-fold in chicks [45]. Although inactivation of the flagellar motor protein MotAB caused *C. jejuni* to be nonmotile, these cells expressed a full-length flagellum yet completely lost the ability to colonize chicks [24, 46]. FlgK is a flagellar hook-associated protein, and the colonization of an *flgK* mutant was five orders of magnitude lower than that observed for wild-type strain in chicks [18]. The flagella secreted *Campylobacter* invasion antigen (Cia) proteins also play a role in colonization. Mutations in CiaI and CiaB lead to a defect in chick colonization compared to the wild-type strain [15, 22, 25]. Furthermore, genes associated with the flagellin O-linked glycosylation system also affect *C. jejuni* colonization and may be mediated by structural and surface changes in the glycan when interacting with the host [16, 23].

Although evaluation of bacterial colonization ability between studies may be difficult due to the diverse methods used by various laboratories, when assayed using the same method the flagellar elements were shown to truly have a various contributions to *C. jejuni* colonization [16]. Thus, the role of FlhF on *C. jejuni* colonization was systematically analyzed in this study. *C. jejuni* is more commonly considered a commensal intestinal microbiota in chickens, where it has a persistent presence in the gut but does not lead to pathology [7]. The chick colonization assay was first performed using both the reference strain NCTC 11168 and an isolate from monkey diarrhea. Different *C. jejuni* strains shows diverse infectivity, and the colonization level of the NCTC 11168 strain was not ideal compared to that of the fecal isolate, which may be due to it being a poor colonizer [9]. In our study, inactivation of *flhF* in NCTC 11168 and the isolate both resulted in significantly decreased *C. jejuni* colonization at 1 and 7 days post inoculation, indicating that the commensal colonization of *C. jejuni* was severely hampered by the loss of *flhF*.

In contrast to chicks, *C. jejuni* infection in humans often leads to a series of illnesses, but the underlying mechanism is not fully understood [47]. To understand whether the importance of *flhF* on commensal colonization was consistent in mammals, mice were used for further assays. The reference strain NCTC 11168 failed to colonize the mice in our assay. Similar findings were also reported in a previous study, where NCTC 11168 was observed to colonize mice at very low efficiency, although after this strain was reisolated from mice it could then colonize mice with a high efficiency [48]. Thus, this phenomenon may be caused by the subculture of this genome-sequenced strain in the laboratory or the need for genetic adaptations in the intestinal tracts to promote its growth [48, 49]. The *C. jejuni* isolate used in our study could stably colonize the assayed mice. Compared to the chicks, the initial establishment of colonization in mice was not severely influenced by the inactivation of *flhF*, which may be due to the diverse characteristics of *C. jejuni* with respect to infecting different hosts. At 7-days post inoculation, all of the isogeneic *flhF* mutants were cleared from the mice, indicating its important role in promoting mice colonization.

The possible factors that may contribute to the colonization defect were investigated. *C. jejuni* growth and metabolism were observed to not be influenced by the mutation of *flhF*, eliminating the possible effects of the *flhF* mutation on bacterial multiplication. Previous studies showed that the motility and flagellar biosynthesis of *C. jejuni* were severely affected by the inactivation of *flhF* [34, 35]. The flagella of *C. jejuni* are involved in not just motility [26], so some of the flagella-associated phenotypes which might play a role in the bacterial colonization were subsequently analyzed. Adherence to the epithelium helps bacteria resist intestinal peristalsis and expulsion [17], and the subsequent invasion is also important. These factors are key determinants for *C. jejuni* colonization [50]. The potential infectivity of the assayed *C. jejuni* strains on Caco-2 cells was assessed. Despite efforts to minimize the influence caused by motility, both the adhered and invaded bacteria were observed to be significantly decreased after inactivation of *flhF*, indicating that the colonization was hampered at this step. Autoagglutination is the basis of bacterial aggregation, which is involved in biofilm formation [51]. Biofilm formation is essential for both environmental survival and successful infections [52, 53], and this mode of bacterial growth is also required for *C. jejuni* colonization [53]. The autoagglutination and biofilm-formation abilities of the *C. jejuni flhF* mutant strain were observed to be decreased in our study, contributing to the observed colonization defect of this strain. The complementation of *flhF* completely or partially restored the defects of adhesion, invasion and biofilm-formation abilities of the *flhF* mutant strain. However, limited restoration of the AAG ability was observed. Previous studies showed that the presence of the flagellin and its glycosylation modification is crucial for *C. jejuni* AAG [51]. We has observed the intact flagellin in the *flhF* complemented strain in our previous study [34], so flagellin glycosylation may not be fully restored after complementation of *flhF*.

In addition to the above mentioned phenotypic changes, the mechanism by which *flhF* affects *C. jejuni* colonization was also explored at the gene expression level. Although *flhF* is a class I flagellar gene that is stably expressed both in vitro and in vivo [34], when the two sets of expression data were compared, we observed that *flhF* is continuously upregulated during the infection process. This observation suggests a close relationship between this gene and *C. jejuni* infectivity, maybe an intact flagellum is required to maintain the secretion of virulence proteins during the infection process [6], or FlhF may function continuously to regulate other virulence factors in an unclarified-method. Inactivation of *flhF* globally affects flagellar gene transcription [34]. Unlike many other flagellated species whose flagellar genes are clustered into operons, the flagellar genes in *C. jejuni* are scattered throughout the genome [34, 54]. Many of these genes are grouped into operons that are involved in other pathways [54], and some genes are also co-expressed with flagellar genes [15]. Thus, we were interested in determining whether *flhF* could affect other infective factors not directly involved in flagellar assembly in this bacterium. Some representative genes were identified based on the literature, and using our previously obtained microarray data [34]. We observed that 45% (39/86) of the colonization and adhesion/invasion related genes were downregulated, among which the expression of flagellar coexpressed determinants (Feds) was apparently affected. This phenomenon may be caused by the Feds genes that belong to the flagellar σ28 regulon [15], which are significantly affected by the inactivation of *flhF* [34].

In this study, we systematically analyzed the influence of FlhF on *C. jejuni* colonization and the possible mechanisms that may contribute to this defect, including the associated virulence phenotypes and a preliminary exploration of changes at the gene expression level. The pathogenesis of *C. jejuni* is not yet well elucidated compared to other analogous species that can also cause diarrheal diseases. *C. jejuni* lacks many of the classic virulence factors [1, 3, 6]. It is generally believed that *C. jejuni* has a regulatory mechanism that co-regulates its virulence. For example, the polar flagella are not only required for motility but also functions as a type III secretion system (T3SS) [26, 54]. The *flhF* gene is continuously upregulated during the infection process and influences the expression of other infection-related factors, which indicates a close relationship between this gene and *C. jejuni* infectivity. It is unknown whether this is an isolated case or a common phenomenon for other flagellar genes or whether other aspects related to *C. jejuni* pathogenesis are affected. These questions still need to be further investigated, which will provide insights into the role of this gene as well as the mechanism of the co-regulation of virulence of *C. jejuni* flagella.

## Conclusion

In this study, the influence of *flhF* on *C. jejuni* colonization was systematically evaluated. Inactivation of *flhF* resulted in severe defects in the commensal colonization of chicks and resulted in a complete loss in the ability of this bacterium to continuously colonize mice. A combination of phenotypic and genetic characterizations revealed that there is a decreased ability of *flhF* mutant strain to adhere and invade epithelial cells, and autoagglutination and biofilm formation, which may contribute to the colonization defect. The *flhF* gene was observed to be continuously upregulated during the infection process and affects the transcription of other infection-related genes that are not directly involved in flagellar assembly, such as the flagellar coexpressed determinants (Feds). This result makes sense, but their definite relationship with *flhF* and the underlying mechanisms that influence *C. jejuni* pathogenesis still requires further investigation.

## Additional file

Additional file 1: **Figure S1.** Identification of the *C. jejuni* isolate. **Table S1.** Primers used in this study. **Table S2.** The relative mRNA expression of other infection-related genes in the microarray assay. **Table S3.** The biochemical characteristics of *C. jejuni* strains. (DOCX 243 kb)

## Abbreviations

AAG: Autoagglutination
ATCC: American Type Cell Culture
CDT: Cytolethal distending toxin
CFU: Colony-forming units
Cia: *Campylobacter* invasion antigen
DMEM: Dulbecco’s minimal essential medium
FBS: Fetal bovine serum
GEO: Gene Expression Omnibus
MH: Mueller-Hinton
NH: Neisseria-Haemophilus
OD: Optical density
PBS: Phosphate buffered saline
qRT-PCR: quantitative real-time polymerase chain reaction
SD: Standard deviation
T3SS: Type III secretion system
