# Supplementary material for: Insights into the impact of flhF inactivation on Campylobacter jejuni colonization of chick and mice gut
Source: BMC Microbiol. 2018 Oct 22;18:149. doi: 10.1186/s12866-018-1318-1 (PMC6196472; doi:10.1186/s12866-018-1318-1)
Supplement: Supplementary file 1 — Figure S1. Identification of the C. jejuni isolate. Table S1. Primers used in this study. Table S2. The relative mRNA expression of other infection-related genes in the microarray assay. Table S3. The biochemical characteristics of C. jejuni strains. (DOCX 243 kb) [file 12866_2018_1318_MOESM1_ESM.docx]

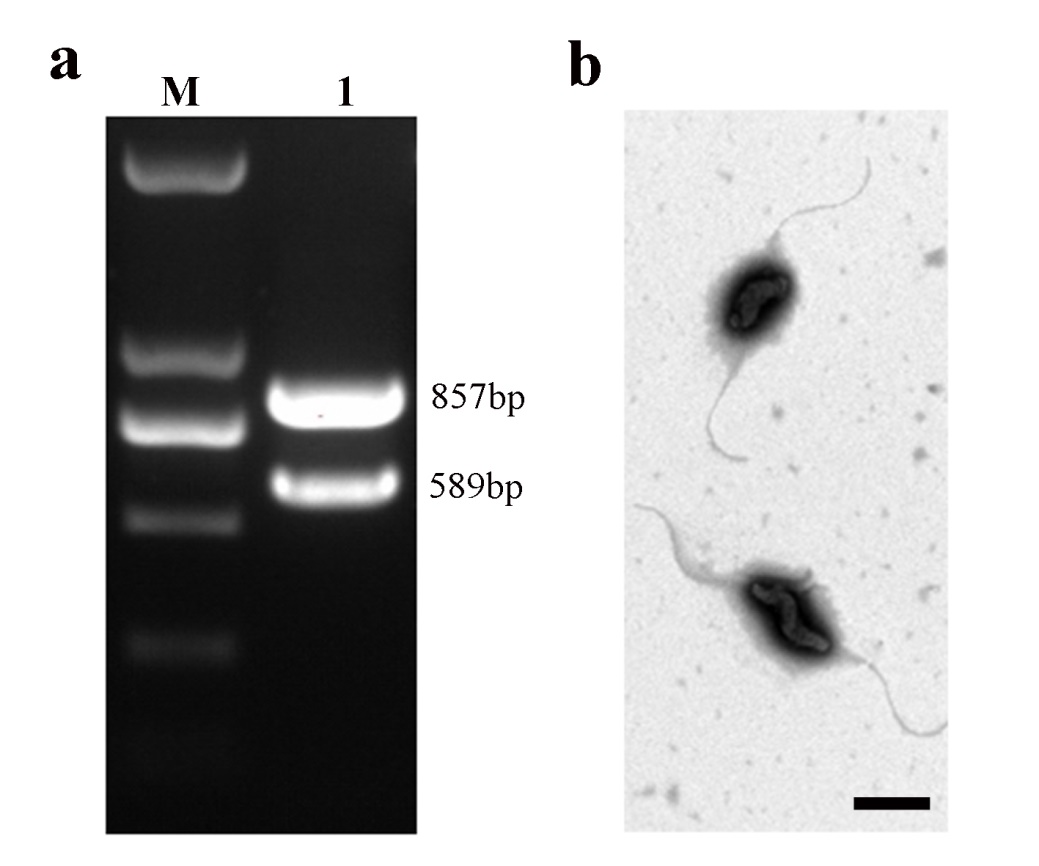


**Fig. S1** Identification of the *C. jejuni* isolate. **a** Multiplex PCR assay was used to identify *Campylobacter* species by primers of 16S rRNA (*Campylobacter* species, 857 bp), *mapA* (*C. jejuni*, 589 bp) and *ceuE* (*C. coli*, 462 bp). Lane M: DNA marker DL2000, Lane 1: PCR profile of the isolate. **b** Transmission electron microscopy of the *C. jejuni* isolate, bars=1 μm.

**Table S1** Primers used in this study

| **Primers** | | **Sequence (5’-3’)** | **Target gene/fragment** |
| --- | --- | --- | --- |
|  | *flhF* RT-F | GATGCTAGTAGGCCCAACAGGA | A fragment of *flhF* |
|  | *flhF* RT-R | AAAGTTGCTCAACTGCACCGA |  |
|  | *cj0402* RT-F | CGATGGAACGGATAATCACC | A fragment of *cj0402* |
|  | *cj0402* RT-R | AATACCTGCATTTCCAAGAGC |  |
|  | *fedD* RT-F | AACGAGGCATTGATGAATGAACT | A fragment of *fedD* |
|  | *fedD* RT-R | GCGTAAATATCCAAACCACTAGAGC |  |
|  | *fedA* RT-F | AAAACCCTACACCTTCAAGAAATCC | A fragment of *fedA* |
|  | *fedA* RT-R | TCTGTGGAACTGCATGGATACG |  |
|  | *fedB* RT-F | AAATACAGCCAATCGCATCGC | A fragment of *fedB* |
|  | *fedB* RT-R | GAGGAAGTTCTCCACCCCACAT |  |
|  | *fedC* RT-F | TTTGAAGTTGACCGCGAAGAG | A fragment of *fedC* |
|  | *fedC* RT-R | TCATTTTGCGGAGTGCATTCT |  |
|  | *aspA* RT-F | AATCGAGGCGACTCAAGATACG | A fragment of *aspA* |
|  | *aspA* RT-R | CATTAAGACCACATTTTGGACCACT |  |
|  | *racS* RT-F | GACGCTTTAATCAATGTTGATATGG | A fragment of *racS* |
|  | *racS* RT-R | CTCACAAGTGCCATTGTTTGAGTAT |  |
|  | *cadF* RT-F | TCTGAAAGACGTGCTAAAAGTGTTG | A fragment of *cadF* |
|  | *cadF* RT-R | TCTGAAAGACGTGCTAAAAGTGTTG |  |
|  | *docC* RT-F | AAAAGCAATGATGAATTTGGACAA | A fragment of *docC* |
|  | *docC* RT-R | ACCACCTTCTACAACTGATACGGTT |  |
|  | *cj0358* RT-F | AATGTAGGACTTGGTGGAACAGATG | A fragment of *cj0358* |
|  | *cj0358* RT-R | CGACCATCCCAAAATTGAGTATTAT |  |

**Table S2** The relative mRNA expression of other infection-related genes in the microarray assay

| **Category** | **Locus tag** | **Gene symbol** | **Proposed function^a^** | **Fold change** | **References** |
| --- | --- | --- | --- | --- | --- |
| **Colonization** | Cj0019c | docB | MCP-domain signal transduction protein | 1.19 | [[1](#_ENREF_1), [2](#_ENREF_2)] |
|  | Cj0020c | docA | cytochrome C551 peroxidase | 1.12 | [[1](#_ENREF_1), [3](#_ENREF_3)] |
|  | Cj0143c | znuA | ABC transporter substrate-binding protein | 1.09 | [[2](#_ENREF_2), [4](#_ENREF_4)] |
|  | Cj0178 | Cj0178 | TonB-denpendent outer membrane receptor | 1.09 | [[2](#_ENREF_2)] |
|  | Cj0248 | Cj0248 | hypothetical protein | 1.10 | [[1](#_ENREF_1)] |
|  | Cj0262c | docC | methyl-accepting chemotaxis signal transduction protein | 0.76 | [[1](#_ENREF_1), [2](#_ENREF_2)] |
|  | Cj0358 | Cj0358 | cytochrome C551 peroxidase | 0.76 | [[2](#_ENREF_2), [3](#_ENREF_3)] |
|  | Cj0368c | cmeR | transcriptional regulator CmeR | 0.94 | [[2](#_ENREF_2)] |
|  | Cj0391c | fedB | hypothetical protein | 0.68 | [[5](#_ENREF_5)] |
|  | Cj0400 | fur | ferric uptake regulator | 1.12 | [[2](#_ENREF_2)] |
|  | Cj0643 | cbrR | two-component response regulator | 1.11 | [[2](#_ENREF_2), [6](#_ENREF_6)] |
|  | Cj0688 | pta | phosphate acetyltransferase | 1.11 | [[1](#_ENREF_1)] |
|  | Cj0883c | Cj0883c | transcriptional regulator | 1.09 | [[1](#_ENREF_1)] |
|  | Cj0903c | Cj0903c | amino acid transport protein | 1.03 | [[1](#_ENREF_1), [2](#_ENREF_2)] |
|  | Cj0923c | cheR | MCP protein methyltransferase | 1.10 | [[2](#_ENREF_2)] |
|  | Cj0924c | cheB | MCP protein-glutamate methylesterase | 1.18 | [[2](#_ENREF_2)] |
|  | Cj0938c | aas | 2-acylglycerophosphoethanolamine acyltransferase | 1.05 | [[1](#_ENREF_1)] |
|  | Cj1018c | livK | branched-chain amino acid ABC transporter substrate-binding protein | 0.87 | [[7](#_ENREF_7)] |
|  | Cj1019c | livJ | branched-chain amino acid ABC transporter substrate-binding protein | 1.12 | [[1](#_ENREF_1), [2](#_ENREF_2), [7](#_ENREF_7)] |
|  | Cj1034c | fedC | adenylosuccinate lyase | 0.72 | [[5](#_ENREF_5)] |
|  | Cj1118c | cheY | chemotaxis protein CheY | 1.11 | [[1](#_ENREF_1), [2](#_ENREF_2)] |
|  | Cj1198 | luxS | S-ribosylhomocysteine lyase | 1.29 | [[2](#_ENREF_2)] |
|  | Cj1222c | dccS | two-component sensor histidine kinase | 1.01 | [[2](#_ENREF_2), [6](#_ENREF_6)] |
|  | Cj1223c | dccR | two-component regulator | 1.10 | [[2](#_ENREF_2), [6](#_ENREF_6)] |
|  | Cj1226c | cprR | two-component sensor histidine kinase | 1.27 | [[2](#_ENREF_2)] |
|  | Cj1227c | cprS | two-component regulator | 1.28 | [[2](#_ENREF_2)] |
|  | Cj1260c | dnaJ | chaperone protein DnaJ | 0.90 | [[2](#_ENREF_2), [8](#_ENREF_8)] |
|  | Cj1261 | racR | two-component regulator | 0.85 | [[2](#_ENREF_2), [6](#_ENREF_6)] |
|  | Cj1262 | racS | two-component sensor histidine kinase | 0.71 | [[2](#_ENREF_2), [6](#_ENREF_6)] |
|  | Cj1355 | ceuE | enterochelin uptake substrate-binding protein | 1.34 | [[2](#_ENREF_2)] |
|  | Cj1398 | feoB | ferrous iron transport protein | 0.86 | [[2](#_ENREF_2)] |
|  | Cj1448c | kpsM | capsule polysaccharide ABC transporter permease | 1.27 | [[2](#_ENREF_2), [9](#_ENREF_9)] |
|  | Cj1506c | tlp1 | MCP-type signal transduction protein | 0.84 | [[2](#_ENREF_2)] |
|  | Cj1614 | chuA | hemin uptake system outer membrane receptor | 1.14 | [[2](#_ENREF_2)] |
|  | Cj1624c | sdaA | L-serine dehydratase | 0.95 | [[10](#_ENREF_10)] |
|  | Cj1656c | fedD | hypothetical protein | 0.47 | [[5](#_ENREF_5)] |
| **Colonization and Adhesion/ invasion** | Cj0045c | fedA | iron-binding protein | 0.53 | [[5](#_ENREF_5)] |
|  | Cj0087 | aspA | aspartate ammonia-lyase | 0.68 | [[11](#_ENREF_11)] |
|  | Cj0169 | sodB | superoxide dismutase | 1.02 | [[2](#_ENREF_2), [11](#_ENREF_11)] |
|  | Cj0454c | Cj0454c | membrane protein | 1.01 | [[1](#_ENREF_1), [11](#_ENREF_11)] |
|  | Cj0456c | Cj0456c | hypothetical protein | 0.98 | [[1](#_ENREF_1), [11](#_ENREF_11)] |
|  | Cj0762c | aspB | aspartate aminotransferase | 0.91 | [[11](#_ENREF_11)] |
|  | Cj0921c | peb1A | bifunctional adhesin/ABC transporter aspartate/glutamate-binding protein | 1.12 | [[2](#_ENREF_2), [12-14](#_ENREF_12)] |
|  | Cj0983 | JlpA | lipoprotein | 1.30 | [[12](#_ENREF_12), [13](#_ENREF_13)] |
|  | Cj1069 | virK | hypothetical protein | 1.03 | [[11](#_ENREF_11), [12](#_ENREF_12), [15](#_ENREF_15)] |
|  | Cj1279c | flpA | fibronectin domain-containing lipoprotein | 1.22 | [[2](#_ENREF_2), [12](#_ENREF_12), [13](#_ENREF_13)] |
|  | Cj1351 | pldA | phospholipase A | 0.99 | [[2](#_ENREF_2), [8](#_ENREF_8), [12](#_ENREF_12)] |
|  | Cj1450 | ciaI | ATP/GTP-binding protein | 0.63 | [[5](#_ENREF_5), [12](#_ENREF_12)] |
|  | Cj1478c | cadF | outer membrane fibronectin-binding protein | 0.73 | [[2](#_ENREF_2), [8](#_ENREF_8), [12](#_ENREF_12), [13](#_ENREF_13)] |
|  | Cj1496c | Cj1496c | periplasmic protein | 1.08 | [[2](#_ENREF_2), [16](#_ENREF_16)] |
| **Adhesion/ invasion** | Cj0039c | typA | GTP-binding protein | 0.93 | [[11](#_ENREF_11)] |
|  | Cj0081 | cydA | cytochrome bd oxidase subunit I | 0.90 | [[11](#_ENREF_11)] |
|  | Cj0091 | Cj0091 | lipoprotein | 0.86 | [[14](#_ENREF_14)] |
|  | Cj0140 | Cj0140 | hypothetical protein | 0.77 | [[11](#_ENREF_11)] |
|  | Cj0190c | Cj0190c | hypothetical protein | 0.96 | [[11](#_ENREF_11)] |
|  | Cj0227 | argD | acetylornithine aminotransferase | 0.88 | [[11](#_ENREF_11)] |
|  | Cj0261c | Cj0261c | SAM-dependent methyltransferase | 0.95 | [[11](#_ENREF_11)] |
|  | Cj0264c | Cj0264c | molybdopterin containing oxidoreductase | 0.83 | [[11](#_ENREF_11)] |
|  | Cj0268c | Cj0268c | transmembrane protein | 1.03 | [[11](#_ENREF_11), [17](#_ENREF_17)] |
|  | Cj0289c | peb3 | major antigenic peptide PEB3 | 1.24 | [[12](#_ENREF_12), [14](#_ENREF_14)] |
|  | Cj0342c | uvrA | excinuclease ABC subunit A | 0.92 | [[11](#_ENREF_11)] |
|  | Cj0587 | Cj0587 | integral membrane protein | 1.02 | [[11](#_ENREF_11)] |
|  | Cj0588 | tlyA | hemolysin | 1.04 | [[14](#_ENREF_14)] |
|  | Cj0596 | peb4cbf2 | peptidyl-prolyl cis-trans isomerase | 0.77 | [[12](#_ENREF_12), [14](#_ENREF_14)] |
|  | Cj0693c | Cj0693c | rRNA small subunit methyltransferase H | 0.72 | [[11](#_ENREF_11)] |
|  | Cj0788 | Cj0788 | hypothetical protein | 0.99 | [[11](#_ENREF_11)] |
|  | Cj0791c | Cj0791c | aminotransferase | 1.00 | [[11](#_ENREF_11)] |
|  | Cj0843c | Cj0843c | transglycosylase | 0.79 | [[11](#_ENREF_11)] |
|  | Cj0859c | FspA | hypothetical protein | 0.69 | [[12](#_ENREF_12)] |
|  | Cj0924c | cheB | MCP protein-glutamate methylesterase | 1.18 | [[11](#_ENREF_11)] |
|  | Cj1068 | Cj1068 | zinc metalloprotease | 0.95 | [[11](#_ENREF_11)] |
|  | Cj1097 | Cj1097 | serine/threonine transporter SstT | 0.85 | [[11](#_ENREF_11)] |
|  | Cj1161c | Cj1161c | cation-transporting ATPase | 1.03 | [[11](#_ENREF_11)] |
|  | Cj1198 | Cj1198 | S-ribosylhomocysteine lyase | 1.29 | [[11](#_ENREF_11)] |
|  | Cj1209 | Cj1209 | ribonuclease Y | 1.09 | [[11](#_ENREF_11)] |
|  | Cj1215 | Cj1215 | peptidase M23 family protein | 1.05 | [[11](#_ENREF_11)] |
|  | Cj1228c | htrA | serine protease | 1.32 | [[11](#_ENREF_11), [12](#_ENREF_12)] |
|  | Cj1242 | ciaC | hypothetical protein | 1.00 | [[12](#_ENREF_12)] |
|  | Cj1249 | Cj1249 | hypothetical protein | 1.10 | [[11](#_ENREF_11)] |
|  | Cj1259 | porA | major outer membrane protein | 0.79 | [[13](#_ENREF_13), [18](#_ENREF_18)] |
|  | Cj1349c | Cj1349c | fibronectin/fibrinogen-binding protein | 1.22 | [[13](#_ENREF_13), [14](#_ENREF_14)] |
|  | Cj1425c | hddA | D-glycero-D-manno-heptose 7-phosphate kinase | 1.40 | [[11](#_ENREF_11)] |
|  | Cj1428c | fcl | GDP-L-fucose synthetase | 0.89 | [[11](#_ENREF_11)] |
|  | Cj1540 | Cj1540 | periplasmic protein | 1.15 | [[11](#_ENREF_11)] |
|  | Cj1647 | iamA | ABC transporter ATP-binding protein | 0.89 | [[12](#_ENREF_12)] |
|  | Cj1685c | bioB | biotin synthase | 1.05 | [[11](#_ENREF_11)] |

a. proposed function are based on the annotated genome sequence from *C. jejuni* NCTC 11168 (Parkhill et al.,2000).

Reference:

[1] Hendrixson DR, DiRita VJ. Identification of Campylobacter jejuni genes involved in commensal colonization of the chick gastrointestinal tract. Molecular microbiology 2004;52:471-84.

[2] Hermans D, Van Deun K, Martel A, Van Immerseel F, Messens W, Heyndrickx M, et al. Colonization factors of Campylobacter jejuni in the chicken gut. Veterinary research 2011;42:82.

[3] Bingham-Ramos LK, Hendrixson DR. Characterization of two putative cytochrome c peroxidases of Campylobacter jejuni involved in promoting commensal colonization of poultry. Infection and immunity 2008;76:1105-14.

[4] Davis LM, Kakuda T, DiRita VJ. A Campylobacter jejuni znuA Orthologue Is Essential for Growth in Low-Zinc Environments and Chick Colonization. J Bacteriol 2009;191:1631-40.

[5] Barrero-Tobon AM, Hendrixson DR. Identification and analysis of flagellar coexpressed determinants (Feds) of Campylobacter jejuni involved in colonization. Molecular microbiology 2012;84:352-69.

[6] Mu X. Identification of Campylobacter virulence and colonisation factors: RMIT University; 2014.

[7] Ribardo DA, Hendrixson DR. Analysis of the LIV system of Campylobacter jejuni reveals alternative roles for LivJ and LivK in commensalism beyond branched-chain amino acid transport. J Bacteriol 2011;193:6233-43.

[8] Ziprin RL, Young CR, Byrd JA, Stanker LH, Hume ME, Gray SA, et al. Role of Campylobacter jejuni potential virulence genes in cecal colonization. Avian diseases 2001;45:549-57.

[9] Jones MA, Marston KL, Woodall CA, Maskell DJ, Linton D, Karlyshev AV, et al. Adaptation of Campylobacter jejuni NCTC11168 to high-level colonization of the avian gastrointestinal tract. Infection and immunity 2004;72:3769-76.

[10] Velayudhan J, Jones MA, Barrow PA, Kelly DJ. L-serine catabolism via an oxygen-labile L-serine dehydratase is essential for colonization of the avian gut by Campylobacter jejuni. Infect Immun 2004;72:260-8.

[11] Novik V, Hofreuter D, Galan JE. Identification of Campylobacter jejuni genes involved in its interaction with epithelial cells. Infect Immun 2010;78:3540-53.

[12] Bolton DJ. Campylobacter virulence and survival factors. Food microbiology 2015;48:99-108.

[13] Flanagan RC, Neal-McKinney JM, Dhillon AS, Miller WG, Konkel ME. Examination of Campylobacter jejuni putative adhesins leads to the identification of a new protein, designated FlpA, required for chicken colonization. Infection and immunity 2009;77:2399-407.

[14] Rubinchik S, Seddon A, Karlyshev AV. Molecular mechanisms and biological role of Campylobacter jejuni attachment to host cells. European journal of microbiology & immunology 2012;2:32-40.

[15] Novik V, Hofreuter D, Galan JE. Characterization of a Campylobacter jejuni VirK Protein Homolog as a Novel Virulence Determinant. Infect Immun 2009;77:5428-36.

[16] Young KT, Davis LM, Dirita VJ. Campylobacter jejuni: molecular biology and pathogenesis. Nature reviews Microbiology 2007;5:665-79.

[17] Tareen AM, Luder CGK, Zautner AE, Gross U, Heimesaat MM, Bereswill S, et al. The Campylobacter jejuni Cj0268c Protein Is Required for Adhesion and Invasion In Vitro. Plos One 2013;8.

[18] Moser I, Schroeder W, Salnikow J. Campylobacter jejuni major outer membrane protein and a 59-kDa protein are involved in binding to fibronectin and INT 407 cell membranes. FEMS microbiology letters 1997;157:233-8.

**Table S3** The biochemical characteristics of *C. jejuni* strains

| **Biochemical reactions/enzymes** | | ***C. jejuni* strains** | | |
| --- | --- | --- | --- | --- |
| Test | Mnemonic | Wild-type | *flhF* mutant strain | *flhF* complementary strain |
| GAMMA-GLUTAMYLTRANSFERAS | GGT | － | － | － |
| L-Lysine-ARYLAMIDASE | LysA | － | － | － |
| D-GALACTOSE | dGAL | － | － | － |
| Leucine-ARYLAMIDASE | LeuA | ＋ | ＋ | ＋ |
| ELLMAN | ELLM | － | － | － |
| Phenylalanine ARYLAMIDASE | PheA | － | － | － |
| L-Proline ARYLAMIDASE | ProA | － | － | － |
| L-Pyrrolydonyl-ARYLAMIDASE | PyrA | ＋ | ＋ | ＋ |
| Tyrosine ARYLAMIDASE | TyrA | － | － | － |
| Ala-Phe-Pro-ARYLAMIDASE | APPA | － | － | － |
| GLYCOGENE | GLYG | － | － | － |
| D-MANNOSE | dMNE | － | － | － |
| D-MALTOSE | dMAL | － | － | － |
| SACCHAROSE/SUCROSE | SAC | － | － | － |
| N-ACETYL-DGLUCOSAMINE | NAG | － | － | － |
| UREASE | URE | － | － | － |
| ORNITHINE DECARBOXYLASE | ODC | ＋ | ＋ | ＋ |
| ALPHA-ARABINOSIDASE | AARA | － | － | － |
| PHOSPHORYL CHOLINE | PHC | － | － | － |
| Phenylphosphonate | OPS | ＋ | － | － |
| PYRUVATE | PVATE | ＋ | ＋ | ＋ |
| Arginine ARYLAMIDASE | ArgA | － | － | － |
| MALTOTRIOSE | MTE | － | － | － |
| L-GLUTAMINE | IGLM | ＋ | ＋ | ＋ |
| PHOSPHATASE | PHOS | － | － | － |
| D-Ribose 2 | dRIB2 | － | － | － |
| D-XYLOSE | dXYL | － | － | － |
| D-GLUCOSE | dGLU | － | － | － |
| BETAGALACTOPRANOSIDASE Indoxyl | BGALi | － | － | － |
| D-MALATE | dMLT | ＋ | ＋ | ＋ |
